# Supplementary material for: Structure of the N-RNA/P interface indicates mode of L/P recruitment to the nucleocapsid of human metapneumovirus
Source: Nat Commun. 2023 Nov 22;14:7627. doi: 10.1038/s41467-023-43434-5 (PMC10665349; doi:10.1038/s41467-023-43434-5)
Supplement: Supplementary file 1 — Supplementary Information [file 41467_2023_43434_MOESM1_ESM.pdf]

Supplementary Information

Structure of the N-RNA/P interface indicates mode of L/P recruitment to the nucleocapsid of human metapneumovirus

Jack D. Whitehead[1,2], Hortense Decool[3], Cédric Leyrat[4], Loic Carrique[1], Jenna Fix[3], Jean-François Eléouët[3], Marie Galloux[3,*], Max Renner[5,6,*]

1 Division of Structural Biology, The Wellcome Centre for Human Genetics, University of Oxford, Oxford, UK

2 Sir William Dunn School of Pathology, University of Oxford, Oxford, UK

3 Université Paris-Saclay, INRAE, UVSQ, VIM, 78350, Jouy-en-Josas, France.

4 Institut de Génomique Fonctionnelle, Université de Montpellier, CNRS, INSERM Montpellier, France

5 Department of Chemistry, Umeå University, Umeå, Sweden

6 Umeå Centre for Microbial Research, Umeå University, Umeå, Sweden

*Corresponding authors: marie.galloux@inrae.fr, max.renner@umu.se

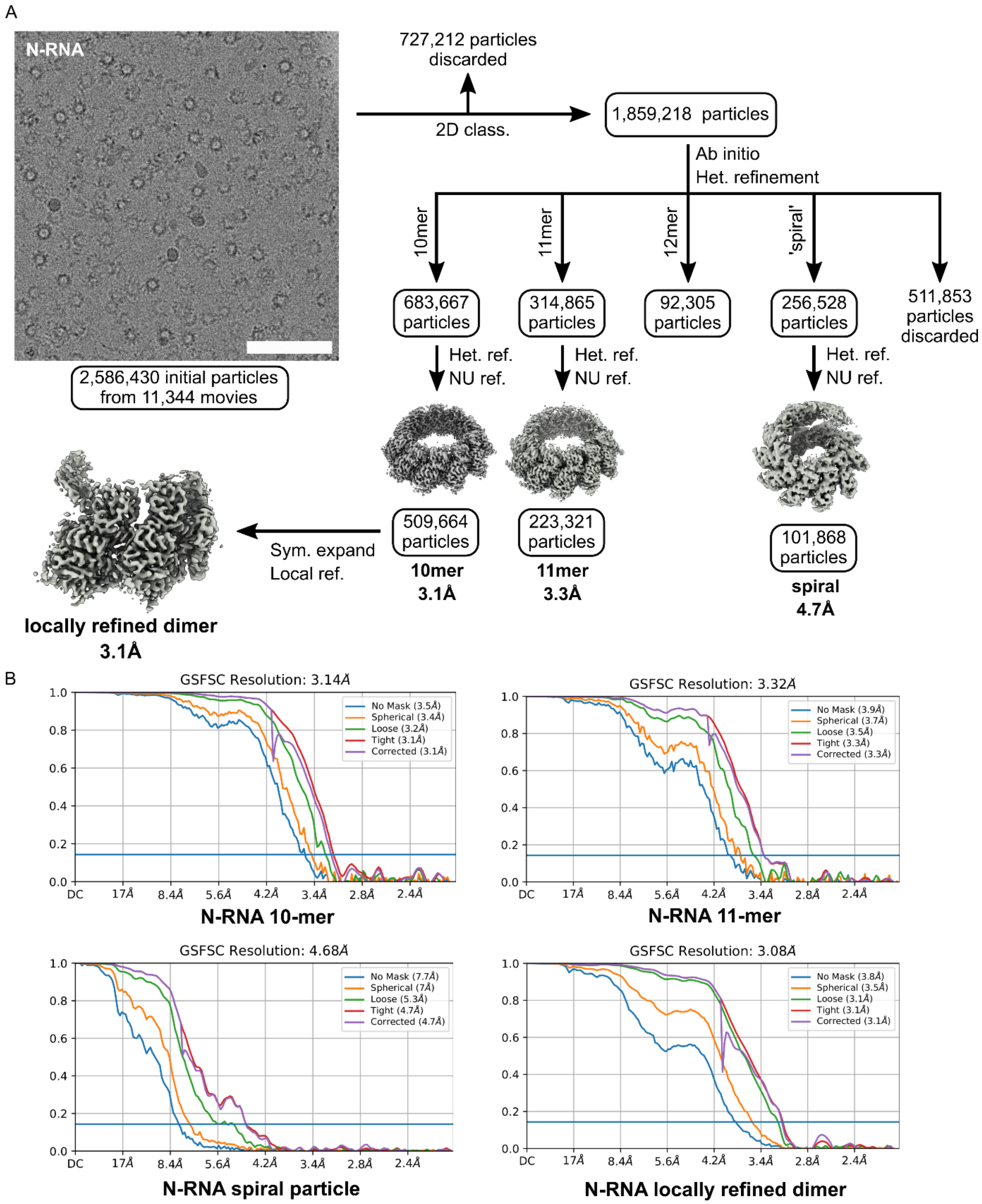

Supplementary Fig. 1: Processing of cryo-EM data of HMPV N-RNA. (A) Processing scheme of the HMPV N-RNA dataset in CryoSPARC. A representative image of the sample is shown on the left. The experiment was repeated 11,344 times with similar results. Scale bar: 75 nm. (B) Fourier shell correlation (FSC) plots of the corresponding reconstructions shown in A.

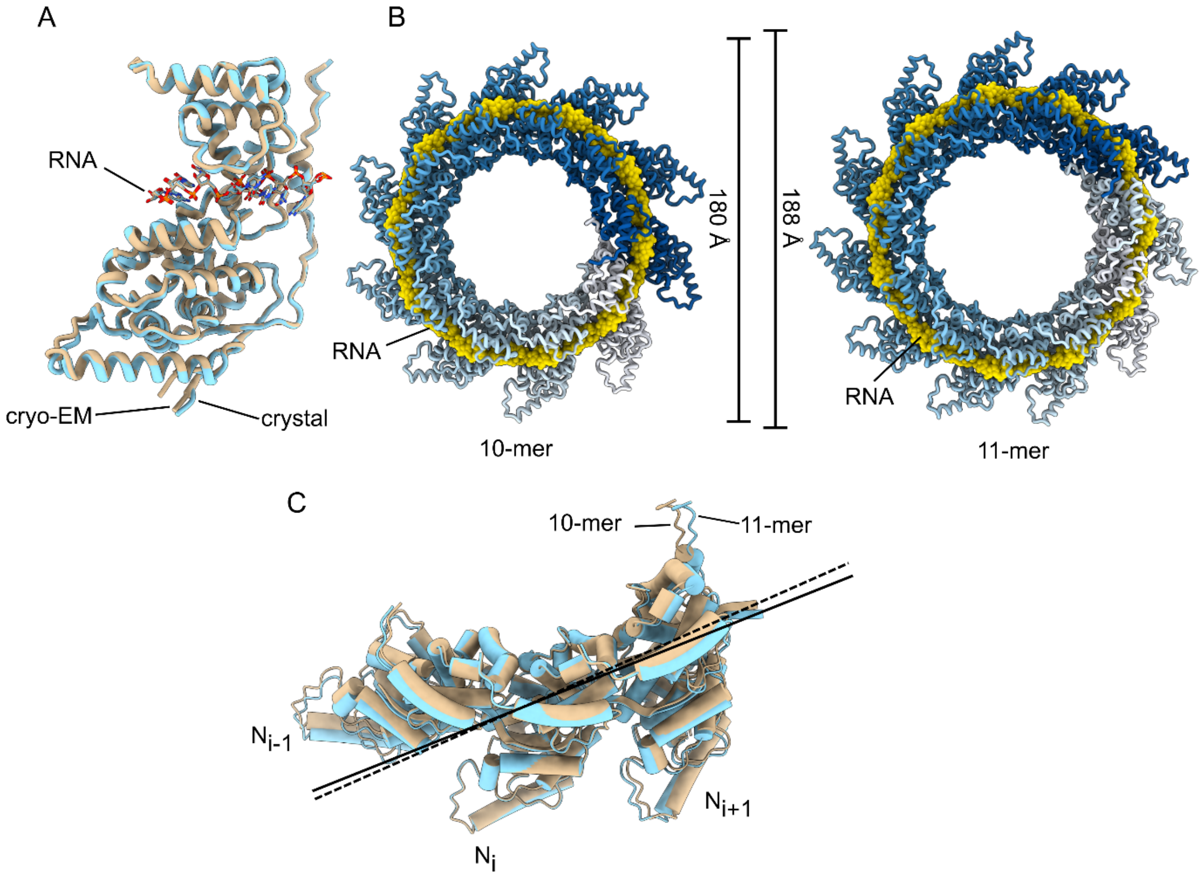

Supplementary Fig. 2: Comparison of N-RNA oligomers. (A) Structural alignment of N-RNA protomers from cryo-EM (this study, beige) and X-ray crystallography (PDB ID: 5FVC, light blue). RNA is shown in stick representation. (B) Comparison of diameter of N-RNA 10-mers and 11-mers (indicated). RNA is shown in yellow. (C) Structural alignment of three neighboring N-RNA protomers from a 10-mer (beige) and 11-mer (light blue). The alignment shows a slightly different angle between neighboring N-protomers from 10-mers and 11-mers, required to accommodate the ring expansion.

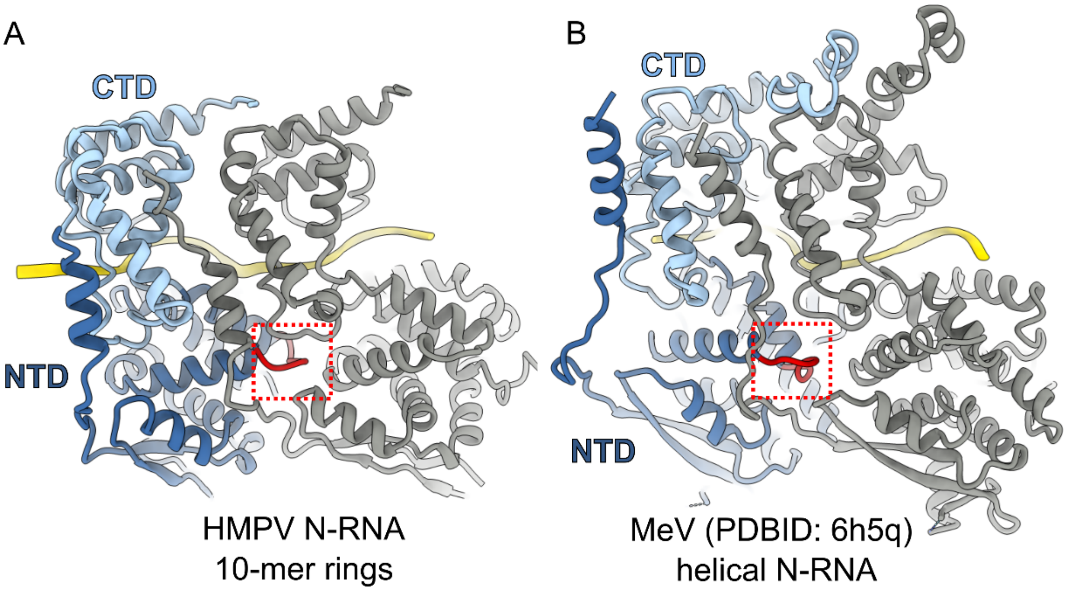

Supplementary Fig. 3: Conservation of lateral loop. (A) Refined model of the HMPV N-RNA dimer from 10-mer rings, viewed from the side opposite of the RNA binding cleft. The lateral loop inserted into a neighboring N-RNA protomer is indicated in red. RNA is shown as a yellow ribbon. (B) Model of two protomers from a helical reconstruction of MeV N-RNA (PDBID 6h5q). Comparison with HMPV N-RNA indicates that the lateral loop is less tightly inserted and forms a less extensive interface with the neighboring protomer, as compared to HMPV (680 Å^2 vs only 395 Å^2 in HMPV and MeV, respectively).

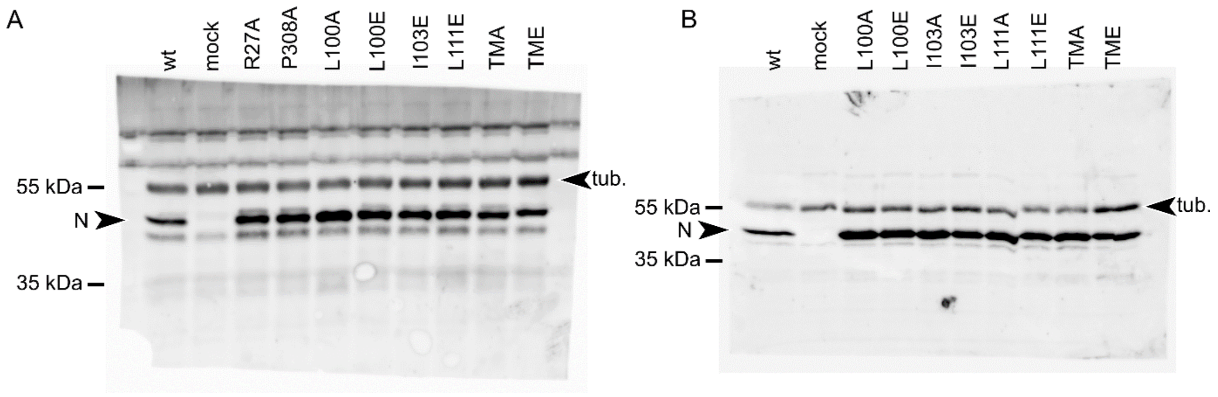

Supplementary Fig. 4: Uncropped Western blots. (A - B) Validation by Western blot of N expression (wt and mutants relative to strain NL/00/1) in BSRT-7 cells transfected in the context of minigenome assays. The heights of N and tubulin bands are indicated. TM-A denotes a triple mutant with alanines in positions 100, 103, and 111. TM-E denotes a triple mutant with glutamates in positions 100, 103, and 111.

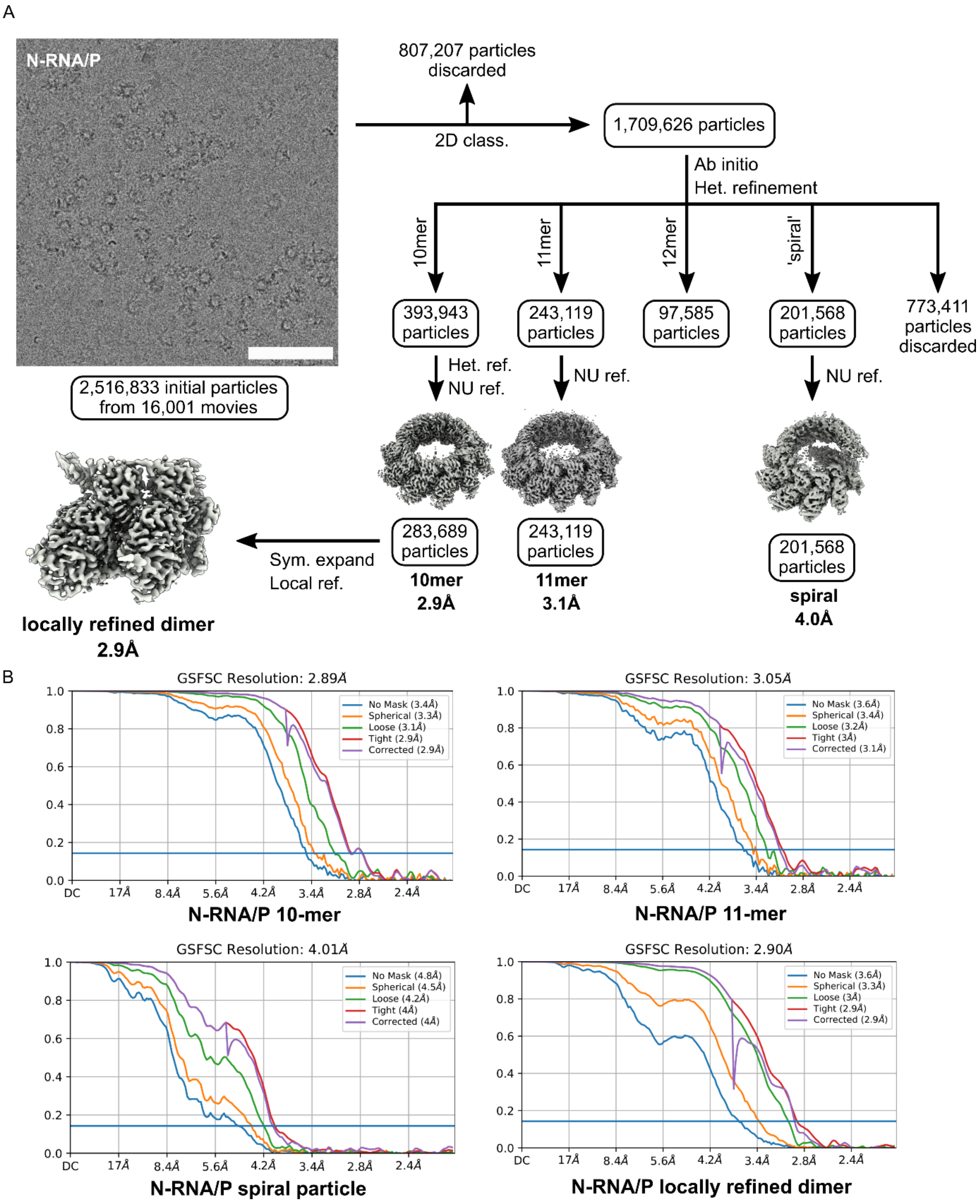

Supplementary Fig. 5: Processing of cryo-EM data of HMPV N-RNA incubated with P_{CT} peptide. (A) Processing scheme of the HMPV N-RNA/P dataset in CryoSPARC. A representative image of the sample is shown on the left. The experiment was repeated 16,001 times with similar results. Scale bar: 75 nm. (B) Fourier shell correlation (FSC) plots of the corresponding reconstructions shown in A.

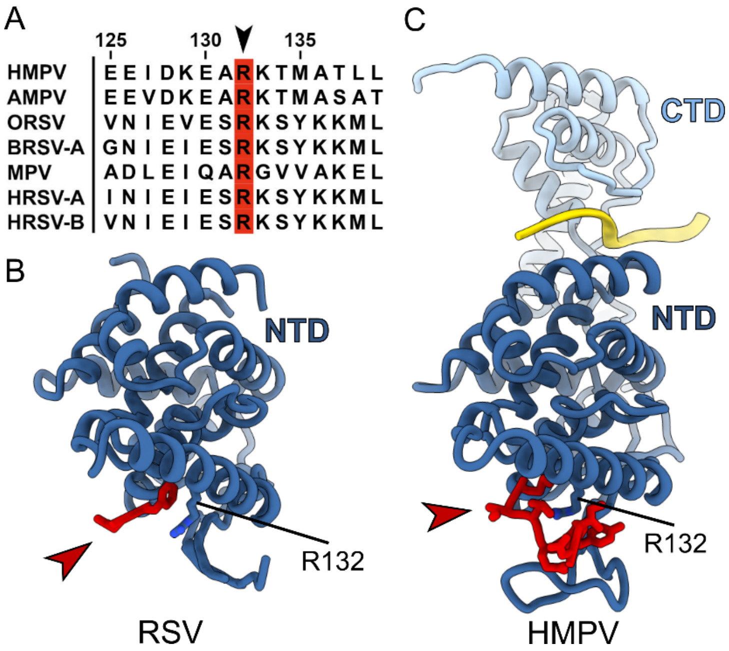

Supplementary Fig. 6: Central role of R132 in P_{CT} attachment in Pneumoviridae. (A) Multiple sequence alignments (MSA) of N sequences from *Pneumoviridae*. A arginine at position 132 is highly conserved (indicated). (B) Structure of the truncated RSV N_{NTD} bound to the last two residues of RSV P (PDBID: 4uc9). Bound P residues are shown in red and are found to pack against R132 (indicated). (C) Structure of one protomer of HMPV N-RNA/P_{CT}. P_{CT} (red) wraps around R132 (indicated). RNA is shown as yellow ribbon.

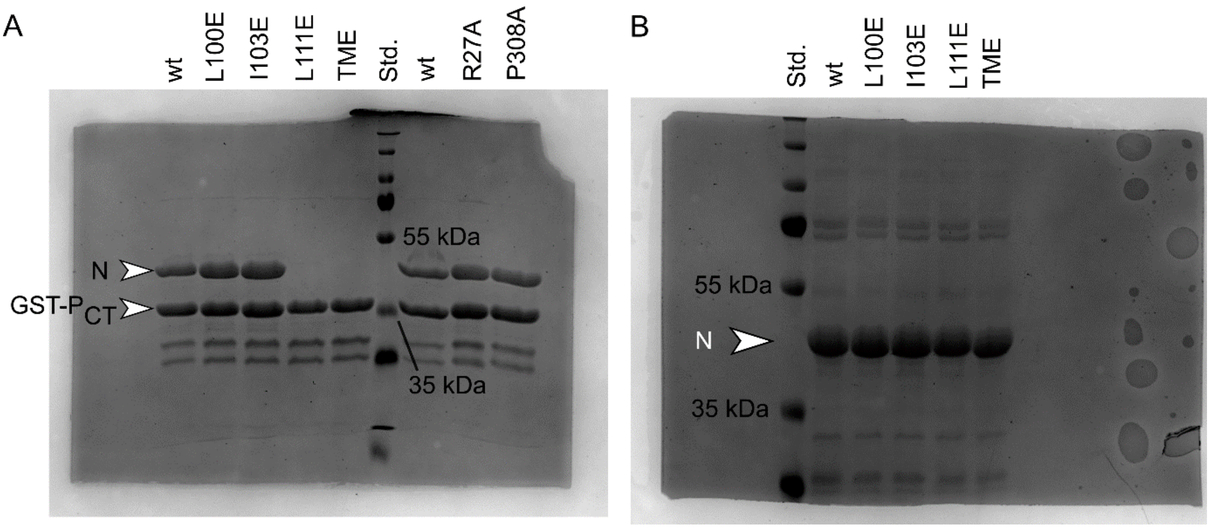

Supplementary Fig. 7: Uncropped SDS-PAGE gels. (A) GST-PCT and 6xHis-tagged N proteins (wt and mutants relative to strain NL/00/1) were co-expressed and co-purified using a GST-tag. A SDS-PAGE analysis of the products of purification is shown, with the position of N and GST-P_{CT} indicated. TM-E denotes a triple mutant with glutamates in positions 100, 103, and 111. (B) SDS-PAGE analysis of N mutants (as in A) purified using 6xHis-tag.

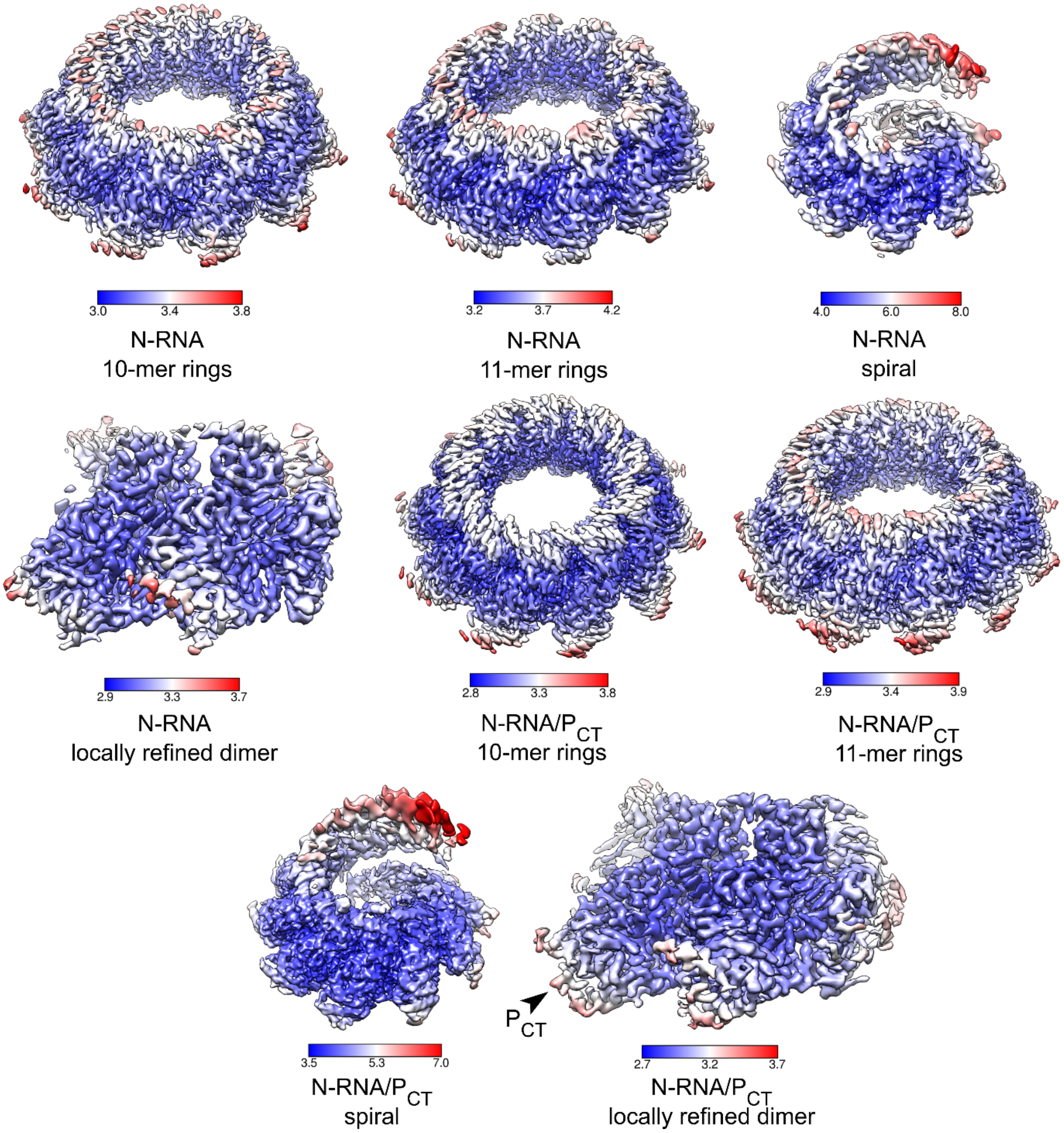

Supplementary Figure 8: Cryo-EM maps of HMPV N complexes colored by local resolution. Maps are colored according to the indicated resolution ranges (in Å) from blue (low resolution) to red (high resolution). The location of a bound P_{CT} is indicated by a black arrow.

Dataset	HMPV N-RNA dataset				HMPV N-RNA/P dataset			
**Structure**	**N-RNA 10-mer**	**N-RNA 11-mer**	**N-RNA Spiral***	**N-RNA dimer Loc. ref.**	**N-RNA/P 10-mer**	**N-RNA/P 11-mer**	**N-RNA/P Spiral***	**N-RNA/P dimer Loc. ref**
**Data collection**								
Microscope	Titan Krios				Titan Krios			
Voltage (kV)	300				300			
Detector	Gatan K2				Gatan K2			
Magnification	130,000x				130,000x			
Pixel size (Å)	1.05				1.05			
Total dose ($e^-/Å^2$)	44.2				44.2			
Def. range (µm)	-1.0 to -2.6				-1.0 to -2.6			
Frames/movie	50				50			
**Data processing**								
Number of movies	11,344				16,001			
Initial particles	2,586,430				2,516,833			
Box size (pixels)	400				400			
Final particles	509,664	223,321	101,868	2,548,320 sym. exp.#	283,689	243,119	201,568	1,418,445 sym. exp.#
Resolution (FSC 0.143)	3.1 Å	3.3 Å	4.7 Å	3.1 Å	2.9 Å	3.1 Å	4.0 Å	2.9 Å
Symmetry	C10	C11	C1	C1	C10	C11	C1	C1
Sharp. B-factor ($Å^2$)	-133	-131	-138	-87	-124	-121	-108	-70
**Model Refinement**								
Composition								
Protein residues	351	351	-	702	370	370	-	744
Nucleotides	7	7	-	14	7	7	-	14
Atoms	2861	2861	-	5722	3016	3016	-	6056
ADP								
Protein	46.39	49.92	-	32.41	39.08	33.95	-	30.52
Nucleotides	26.63	38.20	-	23.51	24.21	21.49	-	17.52
RMSD from ideal								
Bond lengths (Å)	0.008	0.006	-	0.006	0.004	0.004	-	0.004
Bond angles (°)	0.820	0.722	-	0.652	0.570	0.553	-	0.568
Validation								
MolProbity score	1.47	1.52	-	1.45	1.46	1.45	-	1.43
Clashscore	8.75	9.80	-	8.31	8.61	8.28	-	7.83
Rotamer outliers (%)	0.00	0.00	-	0.00	0.00	0.00	-	0.00
CC model vs. map	0.79	0.78	-	0.81	0.80	0.81	-	0.83
Ramachandran								
Favored (%)	98.27	97.98	-	98.13	98.63	98.08	-	98.37
Allowed (%)	1.73	2.02	-	1.87	1.37	1.92	-	1.63
Outliers (%)	0.00	0.00	-	0.00	0.00	0.00	-	0.00
Data Deposition								
PDB	8PDL	8PDM	8PDN	8PDO	8PDP	8PDQ	8PDR	8PDS
EMDB	17613	17614	17615	17616	17617	17618	17619	17620

Supplementary Table 1: Cryo-EM data collection and refinement statistics. Only a single asymmetric unit (ASU) was refined when C10 or C11 symmetry was applied.

*For spiral particles, atomic refinement was not carried out (only rigid-body) due to insufficient map quality.
#Number of particles following from 5-fold symmetry expansion

**Vector**	**Mutations**	**Oligonucleotides**
**pN (minigenome assay)**	R27A+	5' AATAAAAGCCGATGTAGGCACCACAACTGCAG 3'
	R27-	5' GTGTATTGAGACTCTTTTAATATAGCATG 3'
	P308A+	5' AAGACAAAGTGCCAAGGCAGGGC 3'
	P308-	5' AGATGTAAAAGCCCAGATTCAGGACC 3'
	L100A+	5' AACATACTCCGCCGGGAAAGGCAAAAAC 3'
	L100E+	5' AACATACTCCGAGGGGAAAGGCAAAAAC 3'
	L100-	5' TTGGTTAGAACCACCTGAAC 3'
	G103A+	5' CTTAGGGAAAGCCAAAAACAGTAAAGGGG 3'
	G103E+	5' CTTAGGGAAAGAGAAAAACAGTAAAGGGG 3'
	G103-	5' GAGTATGTTTTGGTTAGAACCACCTG 3'
	L100A-G103A+	5' CGCCGGGAAAGCCAAAAACAGTAAAG 3'
	L100E-G103E+	5' CGAGGGGAAAGAGAAAAACAGTAAAGGGG 3'
	L111A+	5' AGGGGAAGAGGCCCAGATGTTAG 3'
	L111E+	5' AGGGGAAGAGGAGCAGATGTTAG 3'
	L111-	5' TTACTGTTTTTGCCTTTCCCTAAGGAG 3'
pET-N	L100E+	5' CGTACTCCGAGGGGAAAGTTAA 3'
	L100-	5' TTCTGGTTAAAACCACTTGGAC 3'
	V103E+	5' GGGAAAGAGAAAAACAACAAAGG 3'
	V103-	5' CAAGGAGTACGTTCTGGTTAAAAC 3'
	L100E-V103E-	5' CTCGGAGTACGTTCTGGTTAAAAC 3'
	L111E+	5' GAGAAGATGAACAGATGTTAGAC 3'
	L111-	5' CTTTGTTGTTTTTAACTTTCCC 3'
	V103E-L111E-	5' CTTTGTTGTTTTTCTCTTTCCC 3'

Supplementary Table 2: Oligonucleotides used in this study.
