# Supplementary material for: Structure of the N-RNA/P interface indicates mode of L/P recruitment to the nucleocapsid of human metapneumovirus
Source: Nat Commun. 2023 Nov 22;14:7627. doi: 10.1038/s41467-023-43434-5 (PMC10665349; doi:10.1038/s41467-023-43434-5)
Supplement: Supplementary file 3 — Description of Additional Supplementary Files [file 41467_2023_43434_MOESM3_ESM.docx]

**Description of Additional Supplementary Files**

**Title:** Supplementary Movie 1

**Description:** Animated 3D rendering of the family of structures from 3D variability analysis (3DVA) in CryoSPARC. 3DVA was carried out with a dimer of HMPV N-RNA and the first variability component showed a prominent transverse tilting motion of N protomers, visualized here as a movie of related maps

**Title:** Supplementary Movie 2

**Description:** Simulated trajectory of a HMPV N-RNA/PCT 5-mer. Snapshots from the MD simulation were taken every nanosecond. N is colored in blue (NTD in dark blue, CTD in light blue) and RNA is colored in yellow. PCT peptides are colored in light red, with one peptide (colored in dark red) unbinding from a N protomer and rebinding at a neighboring protomer in the course of the simulation.
