# Supplementary material for: Structure of the N-RNA/P interface indicates mode of L/P recruitment to the nucleocapsid of human metapneumovirus
Source: Nat Commun. 2023 Nov 22;14:7627. doi: 10.1038/s41467-023-43434-5 (PMC10665349; doi:10.1038/s41467-023-43434-5)
Supplement: Supplementary file 6 — Reporting Summary [file 41467_2023_43434_MOESM6_ESM.pdf]

nature portfolio

Corresponding author(s): Marie Galloux and Max Renner

Last updated by author(s): Oct 31, 2023

Reporting Summary

Nature Portfolio wishes to improve the reproducibility of the work that we publish. This form provides structure for consistency and transparency in reporting. For further information on Nature Portfolio policies, see our Editorial Policies and the Editorial Policy Checklist.

Statistics

For all statistical analyses, confirm that the following items are present in the figure legend, table legend, main text, or Methods section.

n/a	Confirmed	
☐	☒	The exact sample size (*n*) for each experimental group/condition, given as a discrete number and unit of measurement
☐	☒	A statement on whether measurements were taken from distinct samples or whether the same sample was measured repeatedly
☒	☐	The statistical test(s) used AND whether they are one- or two-sided<br>*Only common tests should be described solely by name; describe more complex techniques in the Methods section.*
☒	☐	A description of all covariates tested
☒	☐	A description of any assumptions or corrections, such as tests of normality and adjustment for multiple comparisons
☐	☒	A full description of the statistical parameters including central tendency (e.g. means) or other basic estimates (e.g. regression coefficient) AND variation (e.g. standard deviation) or associated estimates of uncertainty (e.g. confidence intervals)
☒	☐	For null hypothesis testing, the test statistic (e.g. *F*, *t*, *r*) with confidence intervals, effect sizes, degrees of freedom and *P* value noted<br>*Give P values as exact values whenever suitable.*
☒	☐	For Bayesian analysis, information on the choice of priors and Markov chain Monte Carlo settings
☒	☐	For hierarchical and complex designs, identification of the appropriate level for tests and full reporting of outcomes
☒	☐	Estimates of effect sizes (e.g. Cohen's *d*, Pearson's *r*), indicating how they were calculated

Our web collection on statistics for biologists contains articles on many of the points above.

Software and code

Policy information about availability of computer code

Data collection	SERIALEM 3.6
Data analysis	PHENIX 1.18, Coot 0.9.7, cryoSPARC V2.1, cryoSPARC- Live2.15, ChimeraX 1.1, Chimera v1.14, Jalview 2.11.2.0, GROMACS2021, ImageJ 1.53

For manuscripts utilizing custom algorithms or software that are central to the research but not yet described in published literature, software must be made available to editors and reviewers. We strongly encourage code deposition in a community repository (e.g. GitHub). See the Nature Portfolio guidelines for submitting code & software for further information.

Data

Policy information about availability of data

All manuscripts must include a data availability statement. This statement should provide the following information, where applicable:

- Accession codes, unique identifiers, or web links for publicly available datasets
- A description of any restrictions on data availability
- For clinical datasets or third party data, please ensure that the statement adheres to our policy

The coordinates and density maps generated in this study have been deposited in the Protein Data Bank (PDB) and the Electron Microscopy Databank (EMDB) and under accession codes: HMPV N-RNA 10mer (PDB ID: 8PDL, EMD-17613), HMPV N-RNA 11mer (PDB ID: 8PDM, EMD-17614), HMPV N-RNA spiral (PDB ID: 8PDN, EMD-17615), local refinement of a HMPV N-RNA dimer (PDB ID: 8PDO, EMD-17616), HMPV N-RNA/P 10mer (PDB ID: 8PDP, EMD-17617), HMPV N-RNA/P 11mer (PDB ID: 8PDQ, EMD-17618), HMPV N-RNA/P spiral (PDB ID: 8PDR, EMD-17619), local refinement of a HMPV N-RNA/P dimer (PDB ID: 8PDS, EMD-17620). The data underlying Figures 1A, 2F, 4B, 4C, and 5A are provided in the Source Data File. Source data are provided with this paper.

Research involving human participants, their data, or biological material

Policy information about studies with human participants or human data. See also policy information about sex, gender (identity/presentation), and sexual orientation and race, ethnicity and racism.

Reporting on sex and gender	This research did not involve human participants.
Reporting on race, ethnicity, or other socially relevant groupings	This research did not involve human participants.
Population characteristics	This research did not involve human participants.
Recruitment	This research did not involve human participants.
Ethics oversight	This research did not involve human participants.

Note that full information on the approval of the study protocol must also be provided in the manuscript.

Field-specific reporting

Please select the one below that is the best fit for your research. If you are not sure, read the appropriate sections before making your selection.

- [x] Life sciences
- [ ] Behavioural & social sciences
- [ ] Ecological, evolutionary & environmental sciences

For a reference copy of the document with all sections, see nature.com/documents/nr-reporting-summary-flat.pdf

Life sciences study design

All studies must disclose on these points even when the disclosure is negative.

Sample size	For minigenome assays, sample size was determined based on previous studies using the same methodology and shown to be appropriate, see doi:10.1128/JVI.00058-12 or doi:10.1128/JVI.00909-21. For cryo-EM data collection, sample size (i.e, amount of movies collected) was based on previous studies using the same methodology and shown to be appropriate, e.g, https://doi.org/10.1038/s41467-021-21505-9. No further sample size calculations were performed.
Data exclusions	Cryo-EM movies of bad quality (e.g. ice contamination or drift) were excluded prior to cryo-EM processing.
Replication	Minigenome assays were carried out at least two times and in quadruplicates. All attempts to replicate data were successful. Individual data points are shown in the bar graphs (center=mean), together with standard deviations. Protein purifications were carried out once. Immunofluorescence experiments were carried out twice, both times successfully with similar results.
Randomization	N/A for minigenome assays and immunofluorescence as they are cell culture-based assays: each sample contains cells transfected with various plasmids, and they must be identified along the experiment, from transfection to data collection, in order to attribute the results to the correct mix of plasmids.<br>For cryo-EM analysis, refinement was carried out according to gold-standard refinement, which involves random assignment of particles to half-datasets.
Blinding	N/A because predetermined samples and conditions were used throughout the study.

Reporting for specific materials, systems and methods

We require information from authors about some types of materials, experimental systems and methods used in many studies. Here, indicate whether each material, system or method listed is relevant to your study. If you are not sure if a list item applies to your research, read the appropriate section before selecting a response.

Materials & experimental systems

n/a	Involved in the study
☐	☒ Antibodies
☐	☒ Eukaryotic cell lines
☒	☐ Palaeontology and archaeology
☒	☐ Animals and other organisms
☒	☐ Clinical data
☒	☐ Dual use research of concern
☒	☐ Plants

Methods

n/a	Involved in the study
☒	☐ ChIP-seq
☒	☐ Flow cytometry
☒	☐ MRI-based neuroimaging

Antibodies

Antibodies used	Polyclonal rabbit antisera raised against recombinant HMPV N expressed in bacteria were obtained from Dr Galloux's lab. Other antibodies were obtained as follows: mouse monoclonal anti-β-tubulin (Sigma, product number T6199, clone DM1A, lot 115M4796V), anti-mouse IgG coupled to HRP (SeraCare, material number 5450-0011, lot 10430730), anti-rabbit IgG coupled to HRP (SeraCare, material number 5450-0010, lot 10437708), anti-rabbit IgG coupled to Alexafluor-488 (Invitrogen, Catalog # A-11059, lot 682615).
Validation	Polyclonal rabbit antisera against recombinant HMPV N was previously validated: Characterization of the Interaction Domains between the Phosphoprotein and the Nucleoprotein of Human Metapneumovirus. Decool H, Bardiaux B, Checa Ruano L, Sperandio O, Fix J, Gutsche I, Richard CA, Bajorek M, Eléouët JF, Galloux M. J Virol. 2022 Jan 26;96(2):e0090921. doi: 10.1128/JVI.00909-21. Epub 2021 Nov 3.<br><br>Mouse monoclonal anti-alpha-tubulin antibody. Validation statement from the company's website: Anti-a-Tubulin antibody, Mouse monoclonal (mouse IgG1 isotype) is derived from the hybridoma DM1A produced by the fusion of mouse myeloma cells (NS1) and splenocytes from BALB/c mice immunized with purified chick brain tubulin. The isotype is determined by a double diffusion immunoassay using Mouse Monoclonal Antibody Isotyping Reagents, Product Number ISO2.<br><br>Anti-mouse secondary antibody, coupled to HRP. Validation statement from the company's website: affinity purified polyclonal antibody to mouse IgG, both heavy and light chains (whole IgG), made in goat and labeled with horseradish peroxidase. Product has been cross-adsorbed to human serum to minimize cross-reactivity to human immunoglobulin. Product is in liquid form. Each lot is tested to assure specificity and lot-to-lot consistency using an in-house ELISA assay.<br><br>Anti-rabbit antibody, coupled to HRP. Validation statement from the company's website: affinity purified polyclonal antibody to rabbit IgG, both heavy and light chains (whole IgG), made in goat and labeled with horseradish peroxidase. Product is in lyophilized form. Each lot is tested to assure specificity and lot-to-lot consistency using an in-house ELISA assay.<br><br>Anti-rabbit IgG coupled to Alexafluor-488. Validation statement from the company's website: Specificity of secondary antibody was demonstrated by specific detection of the target immunoglobulin. Antibody specificity was demonstrated by specific detection of Mouse IgG. Band at ~55 and 25 kDa corresponding to Mouse IgG Heavy and Light Chain were observed in Mouse IgG but not in other species using Rabbit anti-Mouse IgG (H+L) Cross-Adsorbed Secondary Antibody, Alexa Fluor™ 488 (Product # A-11059) in Western Blot.

Eukaryotic cell lines

Policy information about cell lines and Sex and Gender in Research

Cell line source(s)	BHK-21 cells (clone BSRT7/5) cells were a gift from from the Conzelmann lab, Gene Center - Max von Pettenkofer-Institute of Virology, Munich, Germany. Reference DOI: https://doi.org/10.1128/jvi.73.1.251-259.1999
Authentication	Cell lines were not authenticated
Mycoplasma contamination	The cells were tested negative for mycoplasma
Commonly misidentified lines (See ICLAC register)	No commonly misidentified lines were used.
